# Attitudes Toward Euthanasia and Physician-Assisted Suicide Among Norwegian Palliative Care Physicians

**DOI:** 10.1177/26892820251372012

**Published:** 2025-09-03

**Authors:** Olav Magnus Fredheim, Eva Gravdahl, Ingeborg Skulberg, Morten Magelssen

**Affiliations:** ^1^ Department of Palliative Medicine, Akershus University Hospital, Lørenskog, Norway.; ^2^ Institute of Clinical Medicine, University of Oslo, Oslo, Norway.; ^3^ Centre for Medical Ethics, Institute of Health and Society, University of Oslo, Oslo, Norway.

**Keywords:** euthanasia, palliative care providers, physician-assisted suicide

## Abstract

**Background:**

Data from other countries have indicated that palliative care physicians are more opposed to assisted dying compared with other physicians. However, little is known about reasons for support/opposition among palliative care physicians.

**Objectives:**

To explore Norwegian palliative care physicians’ attitudes toward assisted dying, with focus on filling knowledge gaps regarding reasons for support/opposition.

**Methods:**

The study is a cross-sectional descriptive survey. All 285 members of the Norwegian Association for Palliative Medicine were invited by e-mail to participate. To allow comparison with previous data, most questions and response alternatives were similar to a 2024 study of Norwegian physicians.

**Results:**

Among the 117 respondents opposition toward assisted dying was observed with 85 (73%) strongly disagreeing and 14 (12%) somewhat disagreeing with legalization of physician-assisted suicide for patients with short life expectancy. There was a clear pattern of less support for assisted dying if indications were wider, with only one respondent partially agreeing that assisted dying should be legalized for patients without severe illness who are tired of life and want to die. The most common reason for opposition was assisted dying representing a breach with professional ethics (88 subjects). The most common reason for support was that legalization could provide a safety for patients and next of kin that suffering could be avoided (14 subjects).

**Discussion:**

A large majority of Norwegian palliative care physicians reject the legalization of assisted dying. Among the small minority who support assisted dying, most believe it should be limited to patients with a short life expectancy.

## Key Message

Norwegian palliative care physicians are more strongly opposed to assisted dying than Norwegian doctors in general. Even the supporters are quite restrictive, with most supporters only supporting assisted dying to patients with short life expectancy. Assisted dying conflicting with professional ethics is the main argument against assisted dying.

## Introduction

Whether assisted dying in the form of euthanasia and/or physician-assisted suicide should be legalized is subject to both public and political debate in several countries, including Norway. Even though the attitudes of Norwegian physicians toward assisted dying have shifted slightly in the direction of acceptance from 2016 to 2024, the support among physicians is only approximately one third of that of the general population.^1,2^

Death, dying and management of severe symptoms are central parts of palliative care. In countries where assisted dying has been legalized and implemented its relationship with palliative care varies, ranging from integration and collaboration to outright opposition.^3^ These differences in the relationship are likely to be influenced by culture, legislation and organization of health care.

Because palliative care physicians both are highly competent in end-of-life care and symptom management and will be affected if assisted dying is legalized, their attitude toward legislation is particularly relevant. Even though there is no consensus among palliative care physicians regarding assisted dying, the European Association for Palliative Care holds the position that provision of euthanasia and physician-assisted suicide should not be part of palliative care.^4^ Several previous studies indicate that palliative care physicians and those working within end-of-life care have more often been opposed to assisted dying than other physicians.^5–9^ In a recent study of Swedish palliative care professionals’ attitudes toward assisted dying 38% opposed euthanasia, 36% supported it, and 26% were undecided.^10^ However, there were large interprofessional differences in attitudes, with 80% of physicians opposing euthanasia.

Even though a higher prevalence of opposition toward assisted dying has been demonstrated in palliative care physicians compared with physicians in general, little is known about their reasons for support or opposition.^5–7,10^ Furthermore, previous studies have not provided data on the specific situations in which palliative care physicians who support assisted dying support that it should be available.

The aim of the study was to explore Norwegian palliative care physicians’ attitudes toward euthanasia and physician-assisted suicide, with focus on filling knowledge gaps regarding reasons for support/opposition and regarding in which clinical situations palliative care physicians support/oppose assisted dying.

## Material and Methods

### Study design and population

The study is a cross-sectional descriptive study. All 285 members of the Norwegian Association for Palliative Medicine were invited by e-mail to participate in the study.

### Questionnaire and data collection

Study data were collected by an online questionnaire (Supplementary Data). Invitation to participate and two reminders were sent by e-mail to all members of the Norwegian Association for Palliative Medicine. Because the link included in the invitation was not deactivated after the participant had completed the questionnaire, it was technically possible for a participant to complete the questionnaire more than once. Responses were considered probable duplicates if responses including sociodemographic variables were identical. However, when checking for duplicates, no probable duplicates were identified. Demographic data including work setting, experience in palliative medicine and medical specialty were collected.

In order to allow comparison to previous data, most questions and response alternatives are similar to a 2024 study in a sample of Norwegian physicians.^2^ Response alternatives were either “strongly agree,” “somewhat agree,” “neither agree nor disagree,” “somewhat disagree,” and “strongly disagree” or “yes”, “no,” and “undecided.” For the questions regarding reasons for support or opposition to assisted dying response alternatives were provided, and respondents could select several reasons. In some analyses the respondents are stratified based on having the competence field in palliative medicine. The competence field is the highest level of formal training in palliative medicine in Norway, is issued by the Norwegian Directorate of Health, and is equivalent to an add-on specialty in palliative medicine.

### Statistics

For reference, data from the present study are compared with published data from a recent study where attitudes toward assisted dying were studied in a general sample of Norwegian physicians (national panel).^2^ When the data are compared, the wording of the questions has been the same. Some palliative care physicians might also be part of the general sample.

Differences between groups were analyzed using Pearson’s chi-square (χ^2^) test or Fisher’s exact test, as appropriate. Likert scale responses were treated as ordinal and compared using the Wilcoxon rank-sum test. Analyses were conducted using Stata/SE 18.0 (StataCorp, College Station, TX, USA); *p* < 0.05 was considered statistically significant.

### Research ethics and data protection

Participation was voluntary and based on written information about the aim and content of the study, and about data protection. The study was approved by the Data Protection Officer at the Norwegian Agency for Shared Services in Education and Research (reference 543609). Data were collected using the tool Nettskjema at University of Oslo. Nettskjema provides secure data collection and storage, also ensuring participants’ anonymity. The number and categories of demographic variables were kept to a minimum in order to ensure anonymity.

## Results

### Study population

The study population of 117 subjects represent a response rate of 41%. Overall, 57% were females and 40% of respondents were above 60 years of age (Table 1). Two-thirds were employed in specialist health care, and two-thirds had palliative care as their main occupation. Anesthesiology, oncology, and general medicine were approximately equally frequent specializations with 21%–28% each. Half of respondents had 10 years or more experience in palliative care, and 44% of respondents had achieved the formal competence field in palliative medicine.

**Table 1.** Characteristics of Palliative Care Physicians (*n* **=** 117), Including Subgroup with Formal Competence in Palliative Medicine (*n* **=** 52)

Characteristic *n* (%)	Pall care physicians (*n* **=** 117)	Subgroup with competence field in palliative medicine (*n* **=** 52)
Women	65 (57%)	27 (55%)
Age distribution		
20–29	0 (0%)	0 (0%)
30–39	7 (6%)	0 (0%)
40–49	31 (27%)	8 (16%)
50–59	31 (27%)	13 (26%)
≥60	46 (40%)	29 (58%)
Employment*		
Hospital	82 (70%)	37 (71%)
Primary health care	23 (20%)	7 (13%)
University	13 (10%)	6 (12%)
Other	15 (13%)	10 (19%)
Medical specialty*		
General medicine	33 (28%)	14 (27%)
Anesthesiology	26 (22%)	12 (23%)
Oncology	24 (21%)	13 (25%)
Other	36 (31%)	15 (29%)
Years working in palliative care		
0–2 years	8 (7%)	0 (0%)
3–5 years	18 (15%)	2 (4%)
6–10 years	32 (28%)	10 (19%)
>10 years	58 (50%)	40 (77%)

*Some respondents had to employers or were certified specialists in two fields.

The study sample did not differ significantly from the 2024 national panel in terms of gender distribution (χ^2^(1) = 0.52, *p* = 0.47) or employment setting (χ^2^(2) = 3.14, *p* = 0.21). The national panel had a mean age of 47.4 years (SD 10.7), while 94% of our respondents were aged ≥40 years, suggesting our sample may include more older physicians.

### Attitudes toward legalization

**FIG. 1.**
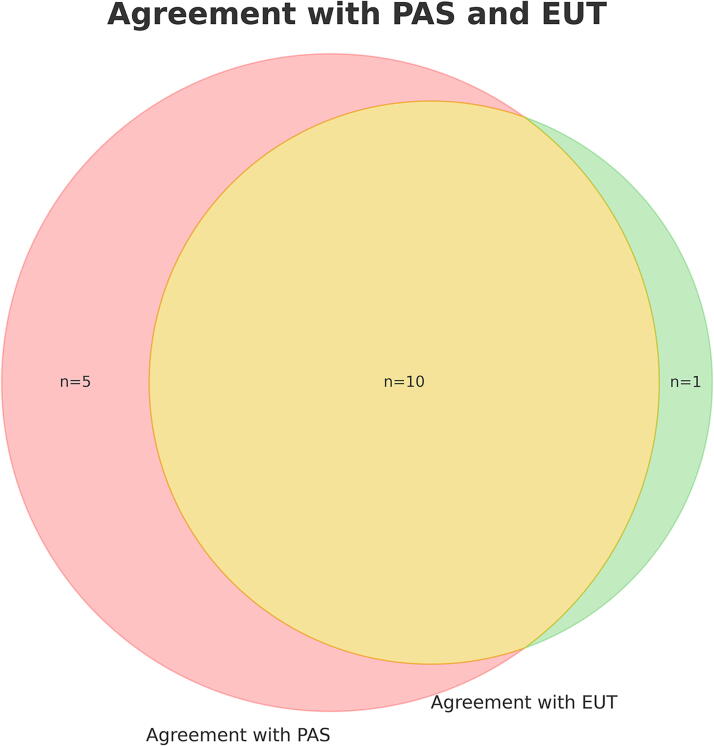
Overlap between support of euthanasia (EUT) and physician-assisted suicide (PAS). Supporters of assisted dying are those “strongly agreeing” or “partially agreeing”.

Among palliative care physicians there is a significant opposition toward assisted dying, with 85 of 117 (73%) strongly disagreeing and 14 (12%) somewhat disagreeing with legalization of physician-assisted suicide for patients with short life expectancy (Table 2). Only 15 of 110 (13%) did either strongly or somewhat agree with such legalization. There was a clear pattern of less support for assisted dying if indications were wider, with only one respondent partially agreeing that assisted dying should be legalized for patients without severe illness who are tired of life and want to die. A significantly higher proportion of palliative care physicians were opposed to assisted dying compared to the national panel of physicians. There was a large overlap between support for euthanasia and support for physician-assisted suicide, but 5 out of 10 who supported physician-assisted suicide did not support euthanasia (Fig. 1).

**Table 2.** Attitudes Toward Assisted Dying Among Norwegian Palliative Care Physicians (*n* **=** 117), with National Comparison of a Norwegian General Sample of Pysicians (*n* **=** 2004)

Statement	Strongly disagree	Somewhat disagree	Neither agree nor disagree	Somewhat agree	Strongly agree	*p* value
Physician-assisted suicide should be permitted for terminally ill patients with a short life expectancy						
Palliative physicians	72.6%	12.0%	2.6%	8.5%	4.3%	<0.001
General sample	36.1%	13.9%	9.8%	26.9%	11.6%	
Euthanasia should be permitted for terminally ill patients with a short life expectancy						
Palliative physicians	81.2%	6.8%	1.7%	6.0%	4.3%	<0.001
General sample	41.2%	15.7%	11.3%	21.1%	9.0%	
Assisted dying (i.e., both physician-assisted suicide and euthanasia) should be permitted for patients with an incurable nonterminal chronic illness						
Palliative physicians	86.3%	8.5%	0.9%	4.3%	0.0%	<0.001
General sample	54.1%	17.7%	12.6%	10.3%	3.7%	
Assisted dying solely due to an incurable mental illness should be permitted						
Palliative physicians	86.3%	8.5%	4.3%	0.9%	0.0%	<0.001
General sample	63.3%	17.4%	11.3%	4.6%	1.7%	
Assisted dying should be permitted for persons who are tired of life and want to die but do not have a serious illness						
Palliative physicians	93.2%	5.1%	0.9%	0.9%	0.0%	*p* = 0.007
General sample	78.1%	9.7%	6.1%	3.3%	1.1%	

### Attitudes toward participation

The numbers of palliative care physicians willing to participate in physician-assisted suicide (12 [10%]) and euthanasia (11 [9%]) are nearly identical (Table 3). However, approximately twice as many are undecided regarding willingness to participate in physician-assisted suicide compared with performing euthanasia. Compared with the national panel of physicians, a significantly smaller proportion were willing to perform assisted dying.

**Table 3.** Willingness to Participate in Assisted Dying, Attitudes Toward Conscientious Objection and Experience with Requests for Assisted Dying Among Palliative Care Physicians (*n* **=** 117)

Statement	Yes	Undecided	No	*p* value
If physician-assisted suicide is legalized, I may be willing to aid this (i.e., by prescribing a lethal drug that the patient takes themselves)				
Palliative physicians	10.3%	12.0%	77.8%	<0.001
General sample	20.5%	23.8%	54.2%	
If euthanasia is legalized, I may be willing to carry it out				
Palliative physicians	9.4%	5.1%	85.5%	0.002
General sample	11.8%	18.0%	68.7%	
If assisted dying is legalized, doctors should have the right to decline (conscientious objection)				
Palliative physicians	95.7%	1.7%	2.6%	0.089
General sample	89.6%	4.9%	3.8%	
Have you as a physician been asked by a patient to perform assisted dying?				
Palliative physicians	64.1%	2.6%	33.3%	Not applicable
General sample	No data			

Data available from a Norwegian general sample of physicians (*n* = 2004) are presented for comparison.

**FIG. 2.**
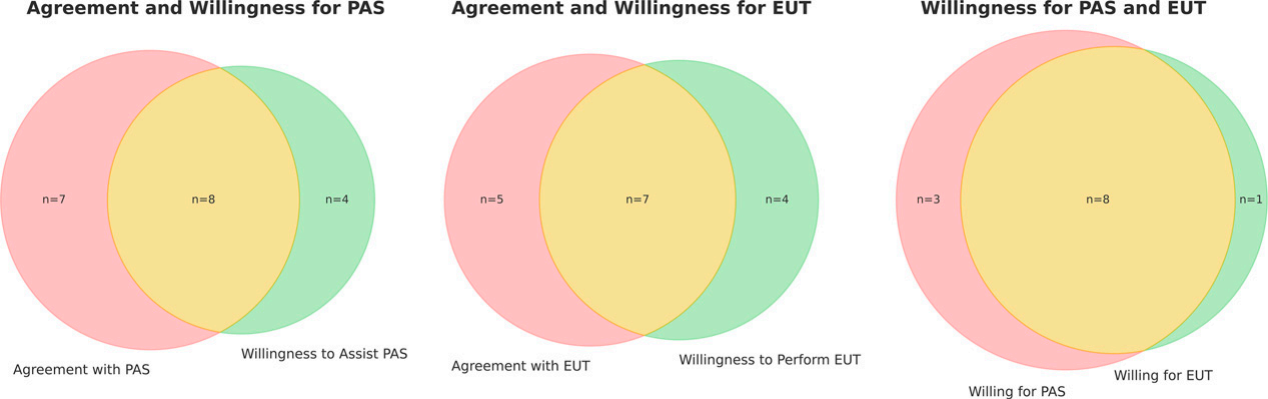
Overlap between support of assisted dying and willingness to participate. Supporters of assisted dying are those “strongly agreeing” or “partially agreeing.”

There is a large degree of overlap between willingness to perform physician-assisted suicide and euthanasia, but less overlap between support for the different forms of assisted dying and willingness to participate (Fig. 2).

### Reasons for opposition and support

On average, participants gave approximately two reasons for either opposition or support to legalization of assisted dying. Twelve participants have given reasons for both support and opposition.

The most common reason for supporting legalization was that legalization could provide a safety for patients and next of kin that suffering could be avoided (14 subjects, 70%). Other frequent reasons were that assisted dying is required as a last resort measure to avoid suffering (11 subjects, 55%) and assisted dying being necessary in order to honor patient autonomy (11 subjects, 55%) (Table 4).

**Table 4.** Reasons for Supporting Assisted Dying

	*N*	%
Euthanasia/physician-assisted suicide is necessary as a last resort for symptom relief	11	55
Euthanasia/physician-assisted suicide is necessary in order to honor patient autonomy	11	55
Euthanasia/physician-assisted suicide is necessary in order to provide health care services in accordance with population preferences	2	10
The availability of assisted dying may provide patients and next of kin reassurance that there is a way to avoid suffering	14	70
I doubt that there in the future will be sufficient access to palliative care, and assisted dying can be an alternative	2	10

A total of 20 respondents, and more than one reason can be given.

The most common reasons for opposition were that assisted dying represents a breach with professional ethics (88 subjects, 81%), assisted dying is not required because symptom control can be achieved with good access to palliative care (81 subjects, 74%), and it would be difficult to achieve a good legislation (72 subjects, 66%) (Table 5).

**Table 5.** Reasons for Opposing Assisted Dying

	*N*	%
Achieving a good legislation would be difficult	72	66
Legalization would represent a breach with the core of professional ethics and the physician role	88	81
Legalization would represent a breach with personal ethical or religious convictions	62	57
Assisted dying is not necessary because effective symptom relief can be achieved, given sufficient access to palliative competence and services	81	74

A total of 109 respondents, and more than one reason can be given.

### Competence field and attitudes

Physicians without the formal competence field in palliative medicine were more likely to support assisted dying for patients with short life expectancy. Among those without the formal competence field, 17% (11/65) somewhat or strongly agreed that physician-assisted suicide should be available, compared to 8% (4/52) of those with the formal competence field in palliative medicine. Similarly, 15% (10/65) of those without the formal competence field supported euthanasia for patients with short life expectancy, while only 4% (2/52) of those with the formal competence field agreed. This difference was statistically significant for both physician-assisted suicide (z = −2.46, *p* = 0.013) and euthanasia (z = −2.71, *p* = 0.007), according to the Wilcoxon rank-sum test.

Physicians with the formal competence field in palliative medicine were less willing to participate in physician-assisted suicide or euthanasia. Specifically, 6% (3/52) of physicians with formal competence were willing to contribute to physician-assisted suicide, compared to 14% (9/65) of those without the formal competence field. Similarly, 4% (2/52) of those with the formal competence field were willing to contribute to euthanasia, while 14% (9/65) of those without competence expressed the same willingness. These differences were statistically significant for both physician-assisted suicide (*p* = 0.042) and euthanasia (*p* = 0.004), based on Wilcoxon rank-sum tests.

### Other findings

A total of 115 (98%) respondents either strongly or somewhat agreed with the statement that there is an ethical distinction between withdrawal of life-prolonging treatment and to perform physician-assisted suicide or euthanasia.

When asked whether their views on assisted dying had changed since starting work as physicians, 63 (54%) reported no change, 29 (25%) had a somewhat more negative view, 11 (9%) had a strongly more negative view, 12 (10%) had a somewhat more positive view, and 2 (2%) had a strongly more positive view.

**FIG. 3.**
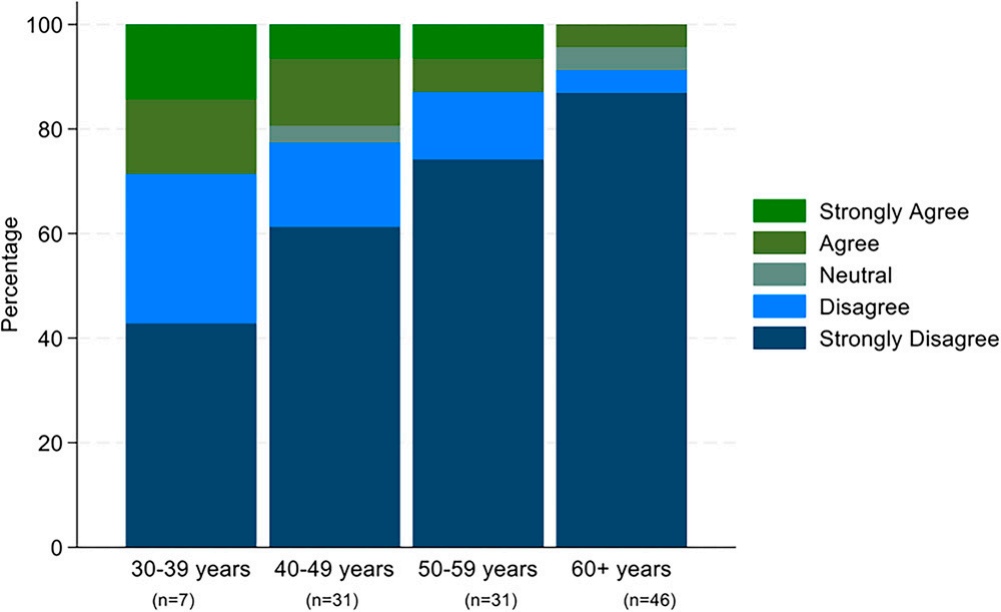
Levels of agreement and disagreement with legalization of physician-assisted suicide for patients with a deadly disease and a short life expectancy stratified by age groups of palliative care physicians.

**FIG. 4.**
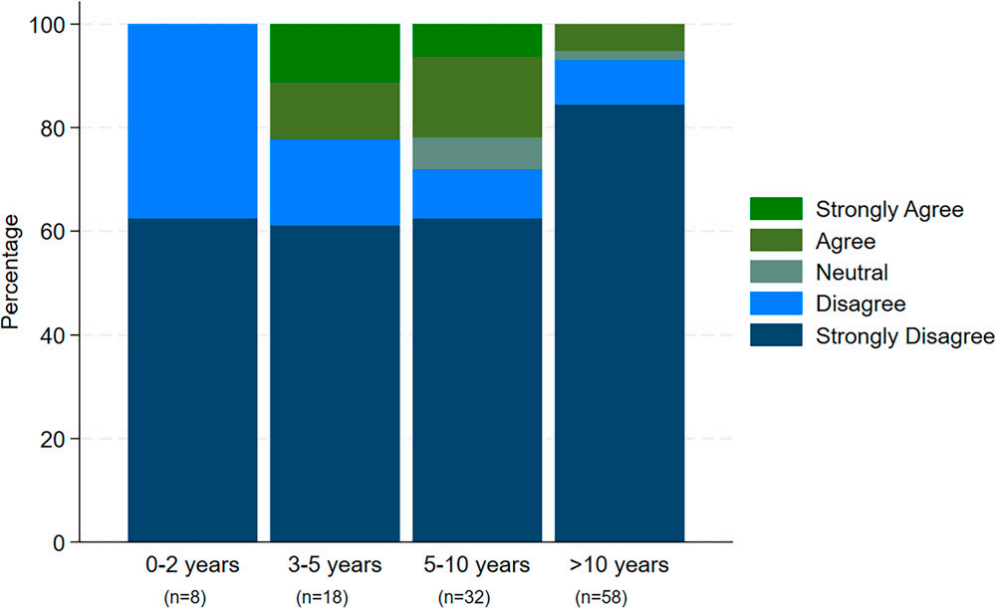
Levels of agreement and disagreement with the legalization of physician-assisted suicide for patients with a deadly disease and short life expectancy, stratified by years of experience among palliative care physicians.

Opposition toward assisted dying is more prevalent among older palliative care physicians compared to younger colleagues, as exemplified with >80% in the age group 60+ strongly disagreeing with legalization of physician-assisted suicide for patients with a deadly disease and a short life expectancy, compared to 40% in the age group 30–39 years (Fisher’s exact *p* = 0.015). (Fig. 3). There is no clear pattern of a relationship between years of experience in palliative care and attitudes toward assisted dying (Fig. 4). Even though the highest proportion of respondents who strongly disagreed was observed in the group with >10 years of experience, all respondents with 0–2 years of experience did either disagree or strongly disagree.

## Discussion

The overall finding in the study is that Norwegian palliative care physicians are more strongly opposed to euthanasia and physician-assisted suicide compared with Norwegian doctors in general.^2^ This finding is in line with previous data and indicates that the physicians with the most extensive clinical experience in treating patients with a high symptom burden at the end of life, have through their clinical experience not been convinced that assisted dying would improve end-of-life care.^5–9^ However, a small minority of palliative care physicians support euthanasia and/or physician-assisted suicide for patients with a deadly disease and short life expectancy. A clear pattern of less support for wider criteria for assisted dying was observed in line with previous studies in different populations.^11,12^

Compared with the general sample of Norwegian doctors, the palliative care physicians are also less willing to perform euthanasia or physician-assisted suicide, and the support for conscientious objection in this area is even higher.^2^ This indicates a strong association between opposition toward euthanasia, support for conscientious objection and unwillingness to perform assisted dying. However, some respondents who did not support assisted dying would nevertheless be willing to assist in physician-assisted suicide or perform euthanasia. It can be speculated that for these respondents the opposition of assisted dying is not rooted in deep religions or ethical convictions, and that they despite their personal opposition would support or at least accept integration between palliative care and assisted dying rather than keeping these services separate. Interestingly, some palliative care physicians who support physician-assisted suicide or euthanasia would not themselves be willing to provide these services. Possible reasons for this constellation of views are a professional view that palliative care and assisted dying should not be integrated, a political view of legalization in conflict with personal values or professional ethics, or an emotional reluctance to taking a patient’s life.

Two thirds of the respondents have been asked by patients to perform euthanasia or physician-assisted suicide. Possibly caring for patients who express a wish for assisted dying could have a strong emotional impact potentially leading palliative care physicians to shift toward supporting assisted dying. However, the opposite was observed. Whereas a slight majority has maintained an unchanged view on assisted dying, three times as many respondents have during their clinical practice become more opposed to assisted dying compared to those becoming more supportive.

The predominant reason for supporting assisted dying was that the availability of assisted dying could provide a sense of safety for patents and next of kin. This line of thinking is in agreement with previous research indicating that patients may see euthanasia as a “solution for the future” if suffering becomes too severe.^13^ The finding that patient autonomy was only in second place among proponents was notable in light of the public and ethical debate where autonomy appears to be a main argument.^14^ The statement that assisted dying should be available in order to meet population preferences for health care services, received minimal support. This indicates that physicians stating autonomy as a reason to offer assisted dying, do not support a “consumerist” conception of health care where there are few or no limits to patient autonomy.

Even though a minority of approximately 15% supported assisted dying for patients with a short life expectancy, assisted dying for patients who do not have a deadly disease received minimal support. This demonstrates that even the supporters are quite restrictive and that they think there should be limits for patient autonomy in this field. By limiting assisted dying to patients with short life expectancy, it appears that palliative care physicians supporting assisted dying would not endorse the broader practice and access to assisted dying implemented in for instance Canada or the Netherlands.

The claim that assisted dying is unnecessary because effective palliation is possible was endorsed by three quarters of respondents who opposed assisted dying. This echoes findings from an interview study with 12 Norwegian palliative care clinicians. Here it was found that refractory suffering was rare according to participants, and that the option of palliative sedation means that adequate symptom control can nearly always be achieved at the very end of life.^15^

Notably, personal ethical or religious convictions were the least common reason for opposition, with only approximately half of respondents giving this reason. This is somewhat surprising given previous reports on strong associations between religious beliefs and opposition toward assisted dying.^11,16,17^ Whereas the finding that many respondents found assisted dying to be in conflict with professional ethics is not surprising, it is surprisingly many who report “difficult to achieve a good legislation” as a reason for opposition. The large diversity in legal criteria for assisted dying, where it is allowed, arguably illustrates that there is no consensus on how legislation in this field could be both clear, fair and safe.^18^ Some respondents gave reasons for both support and opposition, probably because they are undecided about whether to oppose or support legalization.

The overwhelming support for the ethical distinction between withdrawal of life prolonging treatment and assisted dying aligns well with well-recognized definitions.^19^

It can only be speculated why palliative care physicians with the formal competence field in palliative medicine were more opposed to assisted dying. Possible reasons are an effect of higher age or longer clinical experience, a higher formal competence being associated with an opinion that symptom relief can be achieved without assisted dying, or a formative effect of the formal training in palliative medicine. The pattern of older palliative care physicians being more opposed to assisted dying than younger, is in line with previous findings in the general population and a general sample of Norwegian physicians.^2,20^

It has previously been demonstrated that question wording and order may influence responses in surveys on attitudes toward assisted dying.^21^ This is an inherent weakness in all studies addressing such attitudes. However, because question wording and order are similar to the most recent study of attitudes toward assisted dying among Norwegian physicians, comparison between these two surveys is less likely to be influenced by wording and order. Nevertheless, it is a weakness that the questions applied in the study have not been formally validated. Even though it is unlikely that a higher response rate would alter the main findings in the present study, it is possible that palliative care physicians with a more neutral view are underrepresented in the study because invitees with strong opinions could be more likely to respond. Given the likely complexity of reasons for supporting or opposing assisted dying, the underlying attitudes should be further investigated using qualitative methods.

In conclusion the study has demonstrated a strong opposition toward assisted dying among Norwegian palliative care physicians. In the small minority supporting assisted dying the predominant view is that assisted dying should only be available for patients with a short life expectancy.

## Authors' Contribution

O.F., M.M.: Conceived the study and developed the protocol. E.G., I.S.: Contributed to the protocol. E.G.: Collected and managed data and performed statistical analyses. O.F.: Drafted and finalized the manuscript. M.M., E.G., I.S.: Critically revised the manuscript.
