# Supplementary material for: Attitudes Toward Euthanasia and Physician-Assisted Suicide Among Norwegian Palliative Care Physicians
Source: Palliat Med Rep. 2025 Sep 3;6(1):415–23. doi: 10.1177/26892820251372012 (PMC12516118; doi:10.1177/26892820251372012)
Supplement: Supplementary Data [file 26892820251372012_supp_data.docx]

**Attitudes toward assisted dying**

Physician-assisted suicide should be permitted for terminally ill patients with a short remaining life expectancy

Strongly agree

Somewhat agree

Neither agree nor disagree

Somewhat disagree

Strongly disagree

Euthanasia should be permitted for terminally ill patients with a short remaining life expectancy

Strongly agree

Somewhat agree

Neither agree nor disagree

Somewhat disagree

Strongly disagree

Assisted dying (i.e. both physician-assisted suicide and euthanasia) should be permitted for patients with an incurable non-terminal chronic illness

Strongly agree

Somewhat agree

Neither agree nor disagree

Somewhat disagree

Strongly disagree

Assisted dying solely due to an incurable mental illness should be permitted

Strongly agree

Somewhat agree

Neither agree nor disagree

Somewhat disagree

Strongly disagree

Assisted dying should be permitted for persons who are tired of life and want to die but do not have a serious illness

Strongly agree

Somewhat agree

Neither agree nor disagree

Somewhat disagree

Strongly disagree

**Willingness to carry out assisted dying and attitudes to the right to decline (conscientious objection)**

If physician-assisted suicide is legalised, I may be willing to aid this (i.e. by prescribing a lethal drug that the patient takes themselves)

Yes

No

Undecided

If euthanasia is legalised, I may be willing to carry it out

Yes

No

Undecided

If assisted dying becomes legal, physicians should have the right to not participate (conscientious objection)

Yes

No

Undecided

Have you as a physician been asked by a patient to perform assisted dying?

Yes

No

Undecided

**Other questions**

If you support legalization of assisted dying. What are relevant arguments (multiple responses allowed)

Euthanasia/physician assisted suicide is required as a measure of last resort for symptom relief

Euthanasia/physician assisted suicide is required in order to honor patient autonomy

Euthanasia/physician assisted suicide is required in order to deliver health care services in accordance with population preferences

The availability of assisted dying may provide patients and next of kin the safety of an option to avoid suffering

I doubt that there in the future will be sufficient access to palliative expertise, and assisted dying can be an alternative

If you are opposed to legalization of assisted dying. What are relevant arguments (multiple responses allowed)

It would be difficult to achieve a good legislation

It would conflict with the core of being a physician and professional ethics

It would conflict with my personal ethical or religions conviction

Assisted dying is not necessary because – given sufficient access to palliative care – it is possible to provide effective relief

I think there is an ethical difference between withdrawing life prolonging treatment (i.e. ventilator, respiratory support, antibiotics) and to perform euthanasia/physician assisted suicide.

Strongly agree

Somewhat agree

Neither agree nor disagree

Somewhat disagree

Strongly disagree

Has your view on assisted dying changed during your medical career?

Strongly changed in negative direction (more opposed)

Somewhat changed in negative direction

No change

Somewhat changed in positive direction

Strongly changed in positive direction

**Demographics**

Gender : Male Female

Age: 20-29 years 30-39 years 40-49 years 50-59 years >60 years

Employment: Primary health care Specialist health care University Other

Work: Mainly (>50%) palliative care Mainly non-palliative work

Employed in a palliative care/ward/unit: Yes No

Specialist training: Anesthesiology Oncology General medicine Other

Competence field in palliative medicine: Yes No

Years working in palliative care: 0-2 years 3-5 years 5-10 years >10 years
